# Reply to Mr. Atkinson on the Obstetric Practice

**Published:** 1816-10

**Authors:** 


*For the London Medical and Physical Journal.*

### *Reply to Mr. Atkinson on the Obstetric Practice;* by Dr. Kinglake.

IT certainly was my intention not to have protracted the controversy respecting obstetric practice beyond my last communication, as it appeared to me that the subject was exhausted of all that could either interest the theorist or edify the practitioner. But your unyielding disputant, Mr. Atkinson, knowing that he had but very feebly sustained by argument argument the ground which we had gratuitously assumed, for uniform personal attendance during the period of parturition, returns to the charge, first applauding the critical performance of his obstetric co-adjutor, Dr. Merriman, and then resorting to his friend, Mr. Hey, jun. for cases which he vainly imagines must give a *coup de grace* to all further opposition! Nature's powers and resources are not to be thus summarily dealt with. That which has originated in consummate wisdom can neither be deficient in physical aptitude, nor in moral adaptation, to the object meant to be effected. This may be regarded as an axiom bottomed on the very order and necessity of things, the truth of which cannot be controverted by visionary assumption and confident declamation.

Dr. Merriman may be Mr. Atkinson's *demonstrator* of proof, and Mr. Hey, jun. his *reporter* of cases on all obstetric questions; yet the unerring principles of nature will keep their ground, and will administer *in due time* all the aid that may be necessary to the function of parturition, with the utmost *general* precision, so as to preclude all occasion for the assistance of art in natural cases, confining such aid to the rare instances that may present of inseparably mis-shaped *pelves*, and other deviations from the healthy standard, that cannot of necessity frequently occur.

Mr. Atkinson may call on the younger Mr. Hey to report one hundred and fifty unnatural cases out of eight hundred and twenty-seven labours, but the testimony will not weigh a feather in the vast scale of human parturition incessantly obtaining, in which the balance in favour of natural efficiency is struck by *millions* against a numerical proportion that would appear ridiculously dwarfish. Will Mr. Atkinson and his reporter, Mr. Hey, backed by Dr. Merriman, affirm that unnatural cases are occurring in the proportion of *one* in *five*? *One hundred and fifty* in *eight hundred and twenty-seven*, is but little short of that proportion!!! Is Mr. Atkinson prepared to propose this *ratio* as the ground of an obstetric corollary for the universal practice of man-midwifery? Into what delusions does the licentiousness of hypothesis betray its zealous votaries! No errors are so inveterate as those which are taught, they are authorized at the out-set, and become sanctioned and even venerated by adoption and practice.

There is no true science but what is founded in nature, but what emanated from that correct and immutable source. All her operations are simple, comprehensive, and efficient, —objects, indeed, for human admiration, and not for censure and distrust. Imaginary science is replete with danger, it precipitates its fanciful possessor on notions and conclusions that but rarely abide the test of correct inquiry. If due limits be not imposed on the theorist, he will substitute his own *hypothesis* for *realities*, and will go on to state and defend them as so many axiomatic truths. Much of this sort of factitiousness pervades the reasoning and persuasions of the more determined obstetricians of the day. If an objection be made to the popular views of the subject of their art, a *report table* is cited, in which the inadequacies of nature are exhibited in frightful and crying instances, and the opponents of such groundless estimates is contumaciously branded with epithets that savour the reverse of every thing like true science.

Mr. Atkinson authorizes the correctness of his strictures on the objection which I have thought proper to adduce against indiscriminate man-midwifery, by observing that the practice is safe in the hands of men of science and humanity. With due deference to men of *undoubted* science, I feel warranted in averring, that the most reprehensible errors that have been committed within my knowledge in midwifery practice, have been by those who had been the most solicitous candidates for being accredited as persons of superior science; whilst the simple, the modest, and the unassuming practitioner, has had the good fortune to meet with but few or no cases requiring scientific aid, and has deservedly acquired and enjoyed the reputation of being the most successful in midwifery practice. Perhaps Mr. Atkinson may say, that such practitioners did not understand the difficult cases that must have presented to them.—Be it so, but Nature did; and *seasonably* afforded all the relief that was necessary. I have now strongly in my reference several man-midwifery practitioners, not less respectable than aged, who have been, as formerly asserted, in full employ, from thirty to fifty years, and have never had an occasion to use an instrument, and who have merited the confidence and preference of their patients by the distinguished safety and advantage of their practice. There has been, indeed, none of the new-fangled *eclat* attached to the reputation of *bad cases* requiring and receiving scientific and manual aid; but there has been the more solid satisfaction of humanely, patiently, and safely attending the *undisturbed* progress of natural parturition.

I know nothing of Mr. Atkinson's science, nor to what length, or under what circumstances, his zeal for the obstetric art would induce him to interpose his erudite skill, but the following sentence makes it obvious that he does not superabound with the sensibilities worthy of a cultivated and a correct mind. Emboldened by what he calls Mr. Hey's statement, statement, he says, "surely Dr. Kinglake will be more cautious in future how he makes assertions so open to attack, for, were he espousing the cause of truth, and asserting some serious popular error, a recourse to falsehood, either through ignorance or design, would not be very likely to insure success." What does this intemperate vilifying opponent of all reform in obstetric practice rest his insinuation on of my having had "recourse to falsehood?" Is this the language of decorous debate? Is it at all like what a liberal enquirer after truth would have employed? "Falsehood" it may be hoped will never intrude its baneful dastardly machinations into philosophical researches, and it is but just to admit, that the person who suspects the crime, cannot be fairly regarded as a *credible* controversialist.

What I have asserted respecting man-midwifery practitioners in full employ during thirty years, having not met with an unnatural presentation, or at least with any case requiring instrumental aid, is extensively in my power to prove, nay, the period in some instances may be lengthened to forty and even to fifty years. If this assertion should excite Mr. Atkinson's astonishment, it ought not to move his anger, and force from him reflections equally unworthy of the scholar and the gentleman. I have not objected to the fidelity of Mr. Hey's statement, nor to that of Mr. Atkinson's cases, but I might justly suspect the accuracy of their observations and the correctness of their conclusions. If the whole conclave of obstetric practitioners were to affirm that about *one* in *five* cases of human parturition would be *unnatural*, I would strenuously attempt the vindication of Nature's competency from so unjust an imputation on her powers, and would fearlessly avow my persuasion that it could not be, that the notion is monstrous, and at irreconcilable variance with the established and immutable order of things. Human nature is universally the same; certain local modifications and influences may vary the external aspect of circumstances, but vitally and essentially the same fundamental principles exist. Mr. Atkinson's speculations, as well as those of others who may coincide in opinion with him on the subject of the obstetric art, may interpret nature very differently from what she really is; but this is no authority for concluding, that the difference insisted on is a reality, and not a mere fiction of an incorrect imagination. Does Mr. Atkinson think, after he has been baffled in his attempt to establish the indispensable necessity of midwifery practice by the undeniable facts of the African, Asiatic, and American women, safely relying on spontaneous parturition, that the opinion of Camper and White, respecting the configuration of the African scull resembling so much that of the monkey as not to be "capable of solving a geometrical problem," at all decisive or even bearing on the point? The formation of the human scull must be in all situations, for the most part, similar; slight deviations may occur between the inhabitants of different latitudes, as they actually do between those of the same geographical parallels, but not in a degree to influence either the parturine or intellectual function. I have no doubt, that the brain of the African scull (regarding it as the source of intellect) is fully as susceptible, with suitable instruction, of comprehending and "solving a geometrical problem," as that of Mr. White, of Mr. Atkinson, or of any other European, could possibly be. Conclusions built on such untenable grounds are worse than fallacious, they are laughably absurd.

Disdaining all further controversy with a person like Mr. Atkinson, who imputed "falsehood" where his own prejudices and crude notions on one of the most important functions of human life, incapacitates him for comprehending positive truths, I shall here close the discussion with that opponent, assuring others of my readiness to resume it, provided it be entered into for the liberal and humane purpose of investigating and determining what ought to be considered as the legitimate and vindicable scope of the obstetric art.

*Taunton; Aug.* 10, 1816.

